# Anti-Ma2 Encephalitis: An Atypical Case Associated With Klüver-Bucy Syndrome and Hypothalamic Dysfunction

**DOI:** 10.7759/cureus.40816

**Published:** 2023-06-22

**Authors:** Cort Wernz, Lauren Pupa, Ashley Ossimetha, Sahifah Ansari, Fanny Moron

**Affiliations:** 1 Radiology, Baylor College of Medicine, Houston, USA

**Keywords:** anti-ma2 antibody, kluver-bucy syndrome, autonomic dysfunction, paraneoplastic syndromes, autoimmune encephalitis

## Abstract

Anti-Ma2 encephalitis is a rare form of autoimmune encephalitis that has classically been described as a paraneoplastic neurobehavioral disorder due to its association with underlying malignancies. We discuss the case of a 30-year-old female with an exceptionally aggressive presentation of anti-Ma2 encephalitis accompanied by Klüver-Bucy syndrome and hypothalamic dysfunction. Her course was complicated by repeated aspiration events secondary to severe hyperphagia and delays in immunosuppressive treatment due to concerns of infection. The patient’s encephalitis was refractory to multiple immunosuppressive therapies and she ultimately expired before a primary malignancy could be detected and treated.

## Introduction

Anti-Ma2 encephalitis is a rare form of autoimmune encephalitis that has classically been described as a paraneoplastic neurobehavioral disorder due to its association with malignancies in over 90% of cases [1]. It most commonly affects males with testicular or small cell lung cancer, though cases have been reported in females with gynecologic malignancies [2]. Commonly affected areas of the brain include the diencephalon, limbic system, and brainstem [1,2]. The underlying pathophysiology involves intracellular Ma2 antigens and is thought to be primarily T-cell mediated, making treatment with immunotherapies difficult [3]. However, the overall prognosis of anti-Ma2 encephalitis is good if the underlying malignancy can be identified and treated, with over 50% of affected patients showing clinical improvement after tumor treatment [1]. We report the case of a young female patient with an aggressive form of anti-Ma2 encephalitis which presented as Klüver-Bucy syndrome (KBS) and hypothalamic dysfunction.

## Case presentation

A 30-year-old female with a history of seizures and autoimmune encephalitis presented to our hospital with eight months of progressively erratic behavior, memory loss, and weight gain. She was last in a normal state of health two years prior to presentation, at which time she started experiencing occasional seizures, memory loss, and behavioral changes. One year prior to presentation, she was briefly hospitalized at an outside institution where she was diagnosed with thyroid peroxidase (TPO)-positive, NMDA-negative autoimmune encephalitis based on a CSF sample. CSF studies were also negative for herpes simplex virus (HSV) and were otherwise normal at that time. A brain MRI at that time demonstrated T2 hyperintensities in the bilateral hippocampi. She improved after treatment with steroids, intravenous immunoglobin (IVIG), and therapeutic plasma exchange (TPE) and was discharged. 

During the year prior to her initial presentation at our hospital, the patient progressively became erratic and often displayed threatening and aggressive behavior towards caregivers, including verbal abuse and throwing objects. Her short-term memory deteriorated to the point where she struggled to recall conversations that occurred five minutes prior. Additionally, she developed dysphagia and gained 160 pounds over the course of 12 months.

**Figure 1:**
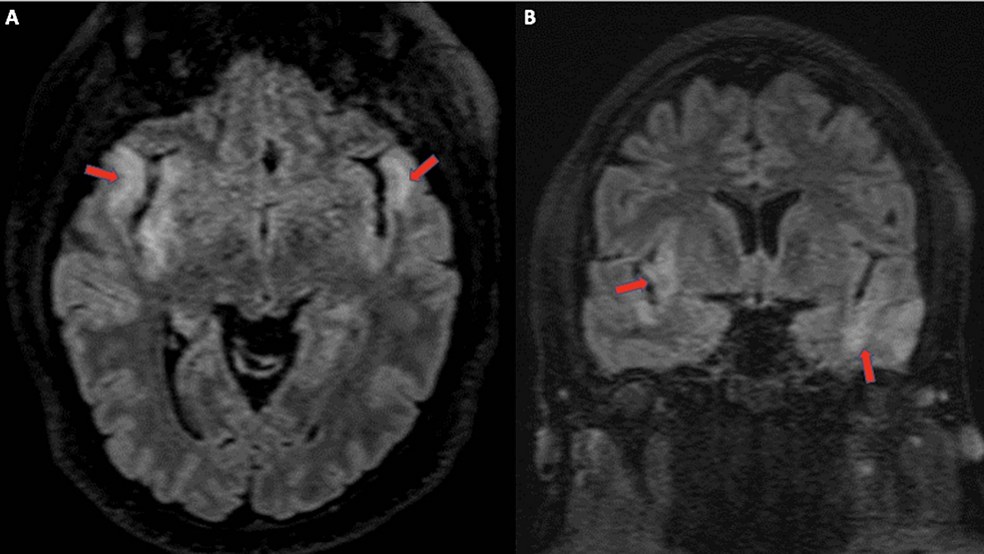
Axial (A) and coronal (B) T2 FLAIR MRI obtained in 2020 Demonstrates hyperintensity of the cortico-subcortical regions of the temporal lobes and right insula (arrows). There was no presence of contrast enhancement or hemorrhage.

A brain MRI was obtained shortly after the initial presentation at our hospital that was notable for worsening cortical and subcortical non-enhancing increased T2 and T2 FLAIR signal in the bilateral temporal lobes, right greater than left (Figures 1A-1B). A lumbar puncture tested negative for HSV and syphilis with no pleocytosis, elevated protein, or oligoclonal bands. TPO antibodies were found to be negative. Multiple subclinical right temporal seizures were noted on continuous EEG which required sedation and intubation for control. She was treated empirically for presumed autoimmune encephalitis with IVIG and steroids with clinical improvement and underwent a repeat MRI which showed an interval improvement of bilateral temporal T2 hyperintensities. She was subsequently discharged home in stable condition with a four-agent anti-seizure drug regimen (sodium valproate, lacosamide, phenytoin, and zonisamide).

**Figure 2:**
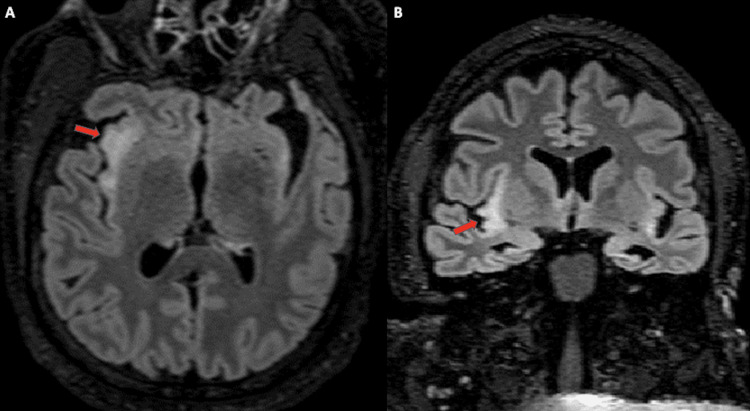
Axial (A) and coronal (B) T2 FLAIR MRI obtained in 2022 Demonstrates persistent hyperintensity of the right insula and new severe atrophy of the temporal lobes/hippocampi (arrows).

Over the course of the following two years, the patient progressively started to exhibit increasingly bizarre behavior including self-urination and defecation, hypersexuality (masturbation, public nudity, sexual solicitation), excessive violence, and worsening hyperphagia. She was re-admitted for further evaluation. A brain MRI showed progressive evolution of abnormal T2 FLAIR hyperintensities in the bilateral temporal lobes and perisylvian regions with hippocampi atrophy compared to the MRI from two years prior (Figures 2A-2B). A lumbar puncture was notable for elevated opening pressure (45 mmHg) but was otherwise unremarkable. A CSF sample was sent to the Mayo Clinic and found to be positive for anti-Ma2 antibodies. 

The patient remained hospitalized for the following five months during which time she was treated with five days of IVIG (400mg/day), five rounds of plasmapheresis, three days of IV steroids (1g/day), two doses of IV rituximab (1000mg/ dose spaced six weeks apart), and one dose of tocilizumab (800mg). Her course was complicated by multiple episodes of aspiration pneumonia due to her hyperphagia. Additionally, her hypersexuality and hyperphagia symptoms persisted during her hospitalization, and she was diagnosed with KBS. A limited malignancy workup, which included multiple CT pan-scans and tumor markers (CEA, CA 19-9, CA, CD20), was negative.

**Figure 3:**
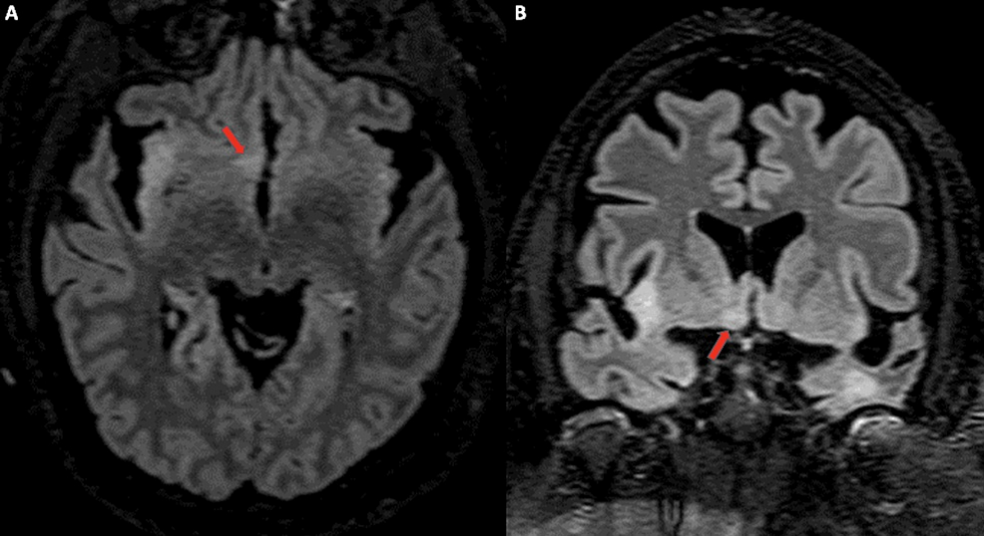
Axial (A) and coronal (B) T2 FLAIR MRI obtained in 2023 Demonstrates new hyperintensity in the right hypothalamus (arrow), persistent hyperintensity in the right insula and left temporal lobe, and severe mostly perisylvian atrophy.

The patient intermittently had fevers as high as 103 °F and low blood pressure, which repeatedly caused delays in immunosuppressive treatment for her autoimmune encephalitis due to concerns for possible infection. Infectious workup including respiratory viral panel, sputum cultures, blood cultures, fungal cultures, urine cultures, and pneumocystis antibody stain came back negative. A repeat brain MRI obtained four months into the hospitalization showed a new T2 FLAIR hyperintensity in the right hypothalamus that was thought to potentially explain her dysautonomia (Figures 3A-3B).

Over the course of her hospitalization, the patient had ongoing seizures despite treatment with lacosamide, topiramate, levetiracetam, and clonazepam that eventually required intubation and sedation with ketamine, midazolam, and phenobarbital for control. Multiple attempts to wean sedation were unsuccessful due to worsening seizure activity. After one month of sedation, the patient’s family decided to transition to comfort care. She was terminally extubated and subsequently expired. The patient’s family denied a request for an autopsy.

## Discussion

Anti-Ma2 encephalitis is a rare form of autoimmune encephalitis that has typically been characterized as a paraneoplastic neurological disorder. The vast majority of affected patients are male and have an associated primary malignancy, with testicular carcinoma being the most common, followed by small cell lung cancer [2]. Our patient exhibited many of the most common features of anti-Ma2 encephalitis including short-term memory loss, cognitive dysfunction, insomnia, agitation, and seizures. T2/FLAIR hyperintensities on brain MRI with temporal lobe involvement have been described in most cases and were present in our patient but may be absent in up to 10% of patients [4]. In addition to our patient being female, there were several features of her presentation that were atypical including the development of KBS, autonomic dysfunction, and undetected malignancy. Unfortunately, due to her severe hospital course and death, further malignancy evaluation was not possible.

KBS is a rare neurological condition that is typically characterized by at least three of the following symptoms: hypersexuality, hyperorality, loss of short-term memory, mutism, seizures, and placidity. KBS can be caused by any infiltrative, degenerative, or infectious insult that damages the bilateral temporal lobes, particularly the hippocampus, amygdala, and adjacent white matter tracts [5]. Our patient exhibited all these symptoms with the exception of placidity and, when combined with bilateral temporal lobe degeneration secondary to her anti-Ma2 encephalitis, met the criteria for diagnosis of KBS. KBS has been reported rarely in some patients with anti-NMDA receptor encephalitis but has not been described in a patient with anti-Ma2 encephalitis [5]. Although some patients with anti-Ma2 encephalitis have been reported to have specific food cravings, such as sugar, none have formally met the criteria for KBS and none have been reported to have the rapid weight gain that was seen in our patient [6].

The anterior hypothalamus is the structure responsible for thermoregulation [7]. In our case, our patient was first noticed to be febrile in December 2022 and continued to have intermittent fever throughout her hospital stay. Despite broad-spectrum antibiotic treatment, escalation of care to the intensive care unit, and extensive fever of unknown origin workups, a physiologic cause for these fevers was never identified. Immunosuppressive therapies were repeatedly interrupted and delayed due to concerns of active infection. Eventually, an MRI brain completed in March of 2023 showed a new hyperintensity in the right hypothalamic region (Figures 3A-3B) that was subsequently hypothesized to be the cause of the patient’s intermittent fevers.

Anti-Ma2 antibody encephalitis affecting the hypothalamus is not uncommon, but our patient’s thermoregulatory issues were. Numerous studies examining anti-Ma2 antibody encephalitis cases in the literature reported MRI brain scans with high-signal intensity in the hypothalamus [8-12]. However, only one of these studies mentions temperature irregularities, with this patient having an axillary temperature of 100.4 °F at admission; there were no other mentions of temperature in this case report [12]. Significant thermoregulatory dysfunction due to dysautonomia from hypothalamic dysfunction should be noted in anti-Ma2 encephalitis to avoid potential delays in immunotherapy.

Lastly, this case report does have some limitations and unanswered questions. Importantly, the anti-Ma2 antibody is associated with a cancer diagnosis in around 90% of cases, and treatment of the underlying malignancy is also the most effective treatment for the associated anti-Ma2 encephalitis [13]. Typically patients positive for this antibody undergo malignancy screening with 18F-fluorodeoxyglucose positron emission tomography (FDG-PET). While this is the most sensitive imaging study for identifying the underlying malignancy, there have been cases of anti-Ma2 encephalitis reported to be associated with carcinoma in situ that is not detectable on PET-FDG [13]. Although our patient was scheduled to undergo an FDG-PET scan in the outpatient setting, she ultimately expired prior to this occurring. While our patient received numerous other inpatient imaging studies that were negative for cancer, an FDG-PET scan may have detected a treatable underlying malignancy that could have altered her course.

## Conclusions

This report describes a case of a patient with anti-Ma2 receptor encephalitis that presented as KBS and symptomatic hypothalamic dysfunction. When the classic symptoms of anti-Ma2 encephalitis are compounded with the hyperphagia characteristic of KBS, patients may be at increased risk for aspiration events. Temperature fluctuations caused by hypothalamic lesions may lead to delays in immunosuppressive therapy due to concerns of infection. Early identification of suspected hypothalamic lesions should be a priority in anti-Ma2 encephalitis patients with intermittent fevers of unknown origin.
